# The impact of vegetation regeneration succession on soil carbon composition and enzyme activity in the southwestern karst region

**DOI:** 10.7717/peerj.21125

**Published:** 2026-04-14

**Authors:** Jianli Zhang, Yanping Li, Tao Pu, Lihua Pu, Yunjie Wu

**Affiliations:** College of Eco-Environmental Engineering, Guizhou Minzu University, Guiyang, China

**Keywords:** Karst ecosystem, Vegetation succession, Soil organic carbon fractions, Soil enzyme activities, Structural equation modeling

## Abstract

Vegetation regeneration enhances soil carbon sequestration in degraded karst ecosystems. However, how succession influences soil organic carbon (SOC) fractions and enzyme activities remains poorly understood in Southwest China’s karst landscapes. We examined three successional stages (grassland, shrubland, and secondary forest) in Pingtang County, Guizhou Province. We quantified SOC fractions, enzyme activities, and their drivers using random forest modeling, partial correlation, and structural equation modeling.SOC content increased significantly from grassland to secondary forest, with forest soils containing 76.74% more SOC than grasslands (*P* < 0.05). Mineral-associated organic carbon dominated all successional stages (51.75–58.81% of total SOC), while microbial biomass carbon increased most during succession (63.84%). Particulate organic carbon remained stable across succession (*P* > 0.05). β-1,4-glucosidase and cellobiohydrolase activities increased with succession, while dehydrogenase and catalase activities decreased. Random forest analysis identified carbon fractions as the dominant predictor of SOC variance (38%). Partial correlation analysis confirmed significant relationships between SOC, carbon fractions, and enzyme activities. Structural equation modeling showed that carbon fractions had the strongest direct effect on SOC accumulation, while soil nutrients had the largest total effect through indirect pathways, regulating enzyme activities and carbon fraction dynamics. These findings elucidate the pathways through which soil nutrients regulate SOC accumulation during karst vegetation succession, advancing understanding of carbon dynamics in degraded karst ecosystems.

## Introduction

Soils store approximately 1,500 Pg of carbon, representing 70% of the terrestrial carbon pool (Yang et al., 2023). This amount exceeds the carbon stored in the atmosphere (650 Pg) and vegetation (700 Pg) combined (Pan et al., 2011; Zhang et al., 2024b). Due to this large reservoir, small changes in soil carbon stocks may considerably alter atmospheric CO_2_ levels and affect global climate (Dixon et al., 1994; Bossio et al., 2020). Vegetation regeneration represents a viable strategy for carbon sequestration and mitigation of anthropogenic emissions (Liu et al., 2015; Bastin et al., 2019). Understanding how vegetation regeneration affects soil carbon sequestration is essential for developing carbon management strategies and enhancing terrestrial carbon sinks.

Soil organic carbon (SOC) is critical for carbon storage, soil fertility, and plant growth, making it a key indicator of soil health (Sollins, Swanston & Kramer, 2007; Wang et al., 2020; Mir et al., 2023). These changes regulate soil physicochemical properties and nutrient cycling, which affect soil organic carbon content (Zhang et al., 2023; Shi et al., 2023). Soil organic carbon consists of multiple fractions with varying stability (Liu et al., 2022). Active organic carbon fractions include dissolved organic carbon (DOC), microbial biomass carbon (MBC), and particulate organic carbon (POC) (Wani, 2021). These fractions have rapid turnover rates and respond sensitively to environmental changes (Wang et al., 2023). Mineral-associated organic carbon (MAOC) represents the inert fraction. Its strong bondingwith minerals ensures slow turnover and stable carbon storage (Shi et al., 2025). During vegetation regeneration, transformations among these carbon fractions determine soil carbon pool size (Ojha et al., 2023). Understanding how vegetation regeneration affects soil carbon fractions is essential for assessing carbon sequestration potential.

Soil extracellular enzymes, as biological catalysts, mediate the transformation of organic carbon components (Xu et al., 2020). Microorganisms and plant roots produce these enzymes, which serve as a critical link in plant-microbe-soil-nutrient systems (Araújo et al., 2013; Burns et al., 2013; Guo et al., 2019). β-1,4-glucosidase (BG) and cellobiohydrolase (CBH) decompose cellulose and related carbohydrates. BG releases glucose, while CBH produces cellobiose for microbial use (Fanin et al., 2022). Dehydrogenase (DHA) participates in organic carbon decomposition and reflects transformation activity (Nannipieri et al., 2012). Catalase (CAT) catalyzes hydrogen peroxide decomposition and correlates with soil redox capacity. Enzyme interactions mediate soil carbon cycling and regulate nutrient availability (Wang et al., 2020). Soil enzyme activity is closely related to SOC, DOC, and MBC, and is also influenced by vegetation and soil pH (Xu et al., 2020). Vegetation affects enzyme activity by regulating litter input and microbial community structure. Soil pH directly affects enzyme activity and modulates catalytic efficiency through its interaction with electrical conductivity. Vegetation regeneration affects soil carbon accumulation by regulating enzyme activity.

The geological structure of the karst areas renders them ecologically fragile, characterized by discontinuous soil distribution, shallow usable soil layers (average thickness <30 cm), high rock exposure rates, and strong heterogeneity in water and soil resources (Wang et al., 2023). This results in extremely high spatial heterogeneity in soil carbon storage. Simultaneously, karst ecosystems exhibit high sensitivity to climate change and human activities, with elevated rates of rock weathering and slow vegetation regeneration (Huang et al., 2021). Studies indicate that karst soil degradation leads to significant reductions in carbon stocks, substantially impacting regional carbon cycling (Breg Valjavec et al., 2022). Consequently, vegetation regeneration in this region holds critical scientific significance for enhancing regional carbon sequestration and conserving ecosystem functions.

At present, research on soil carbon sequestration in karst regions has primarily focused on variations in total soil organic carbon and individual active carbon fractions (Huang et al., 2021; Cheng et al., 2023; Zhang et al., 2024c). However, how carbon fractions and enzyme activities respond to vegetation succession, as well as the regulatory pathways through which soil properties influence SOC accumulation, remain unclear. Therefore, this study focuses on a successional gradient of grassland, shrubland, and secondary forest in the karst regions of Southwest China to quantify the dynamic changes in carbon fractions and enzyme activities and their relationships with soil properties. We hypothesized that (i) SOC content increases along vegetation succession, with differentiated responses among carbon fractions (DOC, MBC, POC, and MAOC), and MAOC represents the dominant component of the soil carbon pool; (ii) the activities of β-1,4-glucosidase and cellobiohydrolase increase during succession, whereas dehydrogenase and catalase activities decline; and (iii) soil nutrients are indirectly associated with SOC accumulation by regulating enzyme activities and carbon fraction dynamics.

## Materials and Methods

### Overview of the research area

**Figure 1:**
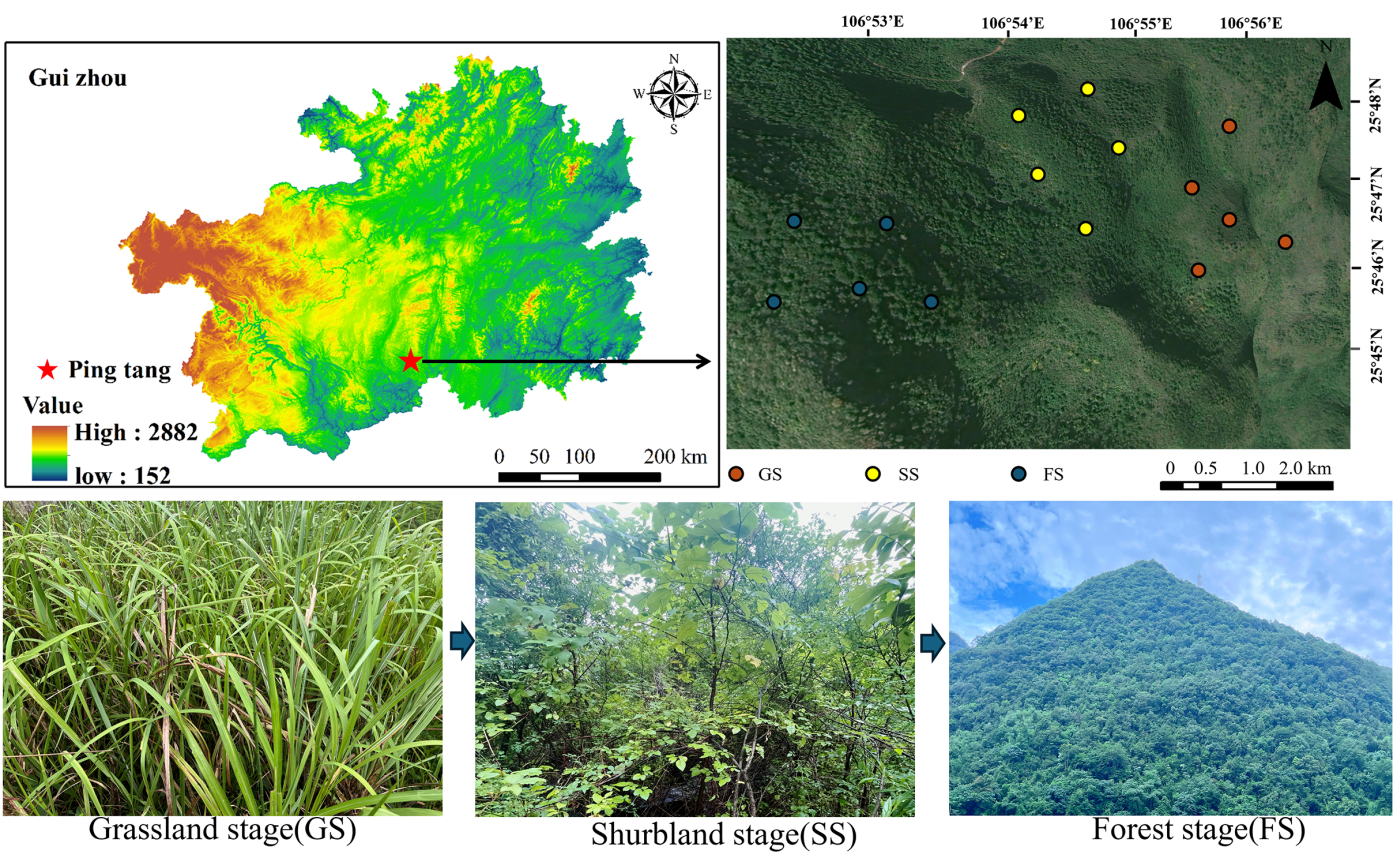
Overview map of the research area.

The study area is located within Kedou Town, Pingtao County, Qiannan Buyi and Miao Autonomous Prefecture, Guizhou Province, China (106°51′02″–106°55′37″ E, 25°45′52″–25°48′13″ N) (Fig. 1). Situated at the transition zone between the Yunnan-Guizhou Plateau and the Guangxi Hills, the area spans elevations of 1,003–1,077 m with a land area of 2,815.60 km^2^. Influenced by a subtropical monsoon climate, it features an annual average temperature of 17 °C, annual precipitation of 1,200 mm, and a frost-free period of 312 days. According to the FAO/UNESCO soil classification system, the soil in the study area is classified as Calcaric Leptosols, a type of calcium-rich shallow soil developed from limestone (Zhang et al., 2022).

### Division of vegetation regeneration succession stages

This study employed a space-for-time substitution approach to examine natural vegetation recovery by comparing plots at different recovery stages. To reduce interference from karst terrain heterogeneity, all plots were selected from areas with Triassic limestone parent material and maintained similar aspects, slopes, and elevations (Table 1). During the 20th century, the study area experienced extensive deforestation and agricultural disturbance, and the timing of human disturbance cessation varied among sites. In 2002, Pingtang County fully implemented the Grain-for-Green Program, resulting in the abandonment of extensive sloping farmlands and a reduction in human disturbance at the regional scale (Shi, 2014). In 2007, human activities further declined following resident relocation associated with the construction of the Five-hundred-meter Aperture Spherical Telescope (FAST) (Li, 2018; Nan & Jiang, 2017). Under comparable environmental conditions, differences in the timing of disturbance cessation formed a temporal gradient corresponding to different stages of vegetation succession. Based on this temporal gradient, three successional stages with distinct recovery histories were selected: grassland stage (GS), shrubland stage (SS), and forest stage (FS). The grassland stage developed from farmlands abandoned in the early 2000s and represents approximately 23 years of natural recovery. Dominant species include *Ficus tikoua*, *Odontosoria chinensis*, *Dicranopteris dichotoma*. The shrubland stage originated from sloping farmlands abandoned in the late 1980s, with an estimated recovery period of 35–40 years. Dominant species include *Cotoneaster fargesii*, *Pyracantha fortuneana*, and *Magnolia multiflora*. The forest stage corresponds to areas where human disturbance ceased before the 1960s, representing more than 60 years of recovery. Dominant species include *Pinus massoniana*, *Quercus acutissima* and *Quercus fabri*.

**Table 1:** Overview of study sites.

Vegetation	Recovery time (year)	Altitude (m)	Slope (°)	Coverage of vegetation (%)	Main plant species	Slope aspect	Disturbance history
Grassland stage	23	1,003 ± 58.24a	37 ± 1.89a	58 ± 1.41c	*Ficus tikoua*	Southwest	Abandoned farmland at the beginning of the 21st century
					*Odontosoria chinensis*		
					*Dicranopteris dichotoma*.		
Shrubland stage	30–35	1,083 ± 59.34a	40 ± 1.66a	79 ± 0.70b	*Cotoneaster franchetii*	Southwest	Sloping farmlands abandoned in the late 1980s
					*Pyracantha fortuneana*		
					*Magnolia multiflora*		
Froest stage	>60	1,077 ± 62.36a	42 ± 2.15a	84 ± 3.32a	*Pinus massoniana*,	Southwest	Natural restoration of forest land on deforested areas before the 1960s
					*Quercus acutissima*		
					*Quercus fabri*		

**Note:**

Data in the table are presented as mean ± standard error. Different lowercase letters indicate significant differences at the *P* < 0.05 level among stages.

### Sample plot layout and soil sample collection

Soil sampling was conducted in mid-November 2024 under normal weather conditions. Fifteen 20 m × 20 m plots were established across three vegetation succession stages–grassland, shrubland, and forest–with five plots per stage. Plots were spaced at least 200 m apart to ensure sample independence. Nine sampling points were arranged in an S-shaped pattern within each plot. After removing surface litter, soil samples were collected from the 0–20 cm layer using a 5 cm diameter soil auger. Rocks, roots, and plant debris were manually removed from each sample and thoroughly mixed to create composite samples for subsequent analysis of physicochemical properties, carbon fractions, and enzyme activities.

### Soil property determination

Soil pH and electrical conductivity (EC) were measured using a soil-water ratio of 1:2.5 in water extracts, determined with a pH meter and conductivity meter, respectively (Li et al., 2025). Soil organic carbon (SOC) was determined using the potassium dichromate oxidation-external heating method (Nelson & Sommers, 1996). Dissolved organic carbon (DOC) was extracted with deionized water by shaking, filtered through a 0.45 μm membrane, and then measured (Jiang et al., 2006). Particulate organic carbon (POC) and mineral-associated organic carbon (MAOC) were physically fractionated after dispersion with sodium hexametaphosphate, followed by potassium dichromate oxidation for carbon content determination (He et al., 2024). Microbial biomass carbon (MBC) was measured using the chloroform fumigation-K_2_SO_4_ extraction method (Vance, Brookes & Jenkinson, 1987). Total nitrogen (TN) was determined by the Kjeldahl method, and total phosphorus (TP) was measured using the molybdenum-antimony colorimetric method (Bao, 2020).

Soil enzyme activities were measured using colorimetric assay kits (Suzhou Michy Biomedical Technology Co., Ltd., Suzhou, China). Air-dried soil samples were passed through a 60-mesh sieve before analysis. For β-1,4-glucosidase (BG; kit No. M1403A), 0.1 g of soil was pretreated with 50 μL toluene for 15 min and incubated with p-nitrophenyl-β-D-glucopyranoside substrate at 37 °C for 1 h. After centrifugation, the supernatant was measured at 405 nm. For cellobiohydrolase (CBH; kit No. M1433A), 0.06 g of soil was incubated with p-nitrophenyl-β-D-cellobioside substrate at 37 °C for 2 h, and the supernatant was measured at 405 nm. For dehydrogenase (DHA; kit No. M1412B), 0.1 g of soil was incubated with 200 μL TTC solution at 37 °C for 24 h, extracted with 1,800 μL methanol, and triphenyl formazan was measured at 485 nm. For catalase (CAT; kit No. M1406B), 0.1 g of soil was incubated with 1,000 μL H_2_O_2_ substrate at 25 °C for 20 min, and residual H_2_O_2_ in the supernatant was measured at 240 nm using a quartz cuvette. All measurements were performed in triplicate with substrate and soil blanks as controls. Enzyme activities were calculated using standard curves and expressed as nmol·g^−1^·h^−1^.

### Statistical analysis

Statistical analysis was performed using SPSS 25.0 and R 4.3.0 software. Shapiro-Wilk normality tests and Levene’s tests for homogeneity of variance were conducted on variables to ensure compliance with parametric test assumptions. One-way analysis of variance (ANOVA) was used to compare soil physicochemical properties, carbon components, and enzyme activities across different vegetation succession stages. Tukey’s HSD multiple comparison test identified significant differences between treatments (*P* < 0.05). For variables not meeting normality assumptions, the Kruskal-Wallis nonparametric test was applied.

To identify key drivers of SOC accumulation and their regulatory mechanisms, random forest analysis was employed to assess variable importance, while path analysis was used to test hypothesized causal pathways based on *a priori* assumptions. The *rfPermute* package in R was used to implement the random forest permutation approach (1,000 permutation iterations) to determine the primary drivers affecting SOC and to quantify the relative importance of soil carbon fractions (MBC, DOC, POC, and MAOC), nutrients (TN and TP), environmental factors (pH and EC), and enzyme activities (BG, CBH, DHA, and CAT). The stability of model predictive performance was assessed using leave-one-out cross-validation (LOOCV), with the coefficient of determination (R^2^) serving as the evaluation metric (Suleymanov et al., 2023). Spearman correlation analysis was conducted using the *psych* package to assess relationships between each category of drivers and SOC. Partial correlation analysis was subsequently performed using the *ppcor* package to further identify key factors independently associated with SOC. Based on widely reported ecological mechanisms and preliminary analytical results, partial least squares path modeling (PLS-PM) was constructed using the *plspm* package to explore the direct and indirect effects of soil carbon fractions, nutrients, environmental factors, and enzyme activities on SOC. Compared with covariance-based structural equation modeling, PLS-PM does not rely on multivariate normality assumptions and is suitable for small sample sizes and situations involving multicollinearity among predictor variables (Carrascal, Galván & Gordo, 2009). Based on widely reported ecological and mechanistic relationships and preliminary analytical results, the hypothesized causal structure paths of the PLS-PM were developed (Shi et al., 2023; Zeng et al., 2024a; Zhang et al., 2024a). The path model was constructed based on the following theoretical hypotheses: (1) environmental factors influence soil nutrients; (2) soil nutrients are influenced by environmental factors and affect soil enzyme activities; (3) soil carbon fractions are jointly influenced by environmental factors, nutrients, and enzyme activities; and (4) SOC is jointly influenced by environmental factors, nutrients, enzyme activities, and carbon fractions. The goodness-of-fit index (GoF) was used to assess the overall model fit of the PLS-PM (Dunn et al., 2021). The coefficient of determination (R^2^) was used to quantify the proportion of variance explained in endogenous variables. Path coefficients and their statistical significance were estimated using bootstrap resampling (1,000 iterations).

## Results

### Soil physicochemical properties and nutrient status in different successional stages

#### Changes in soil physical and chemical properties and carbon fractions content

**Figure 2:**
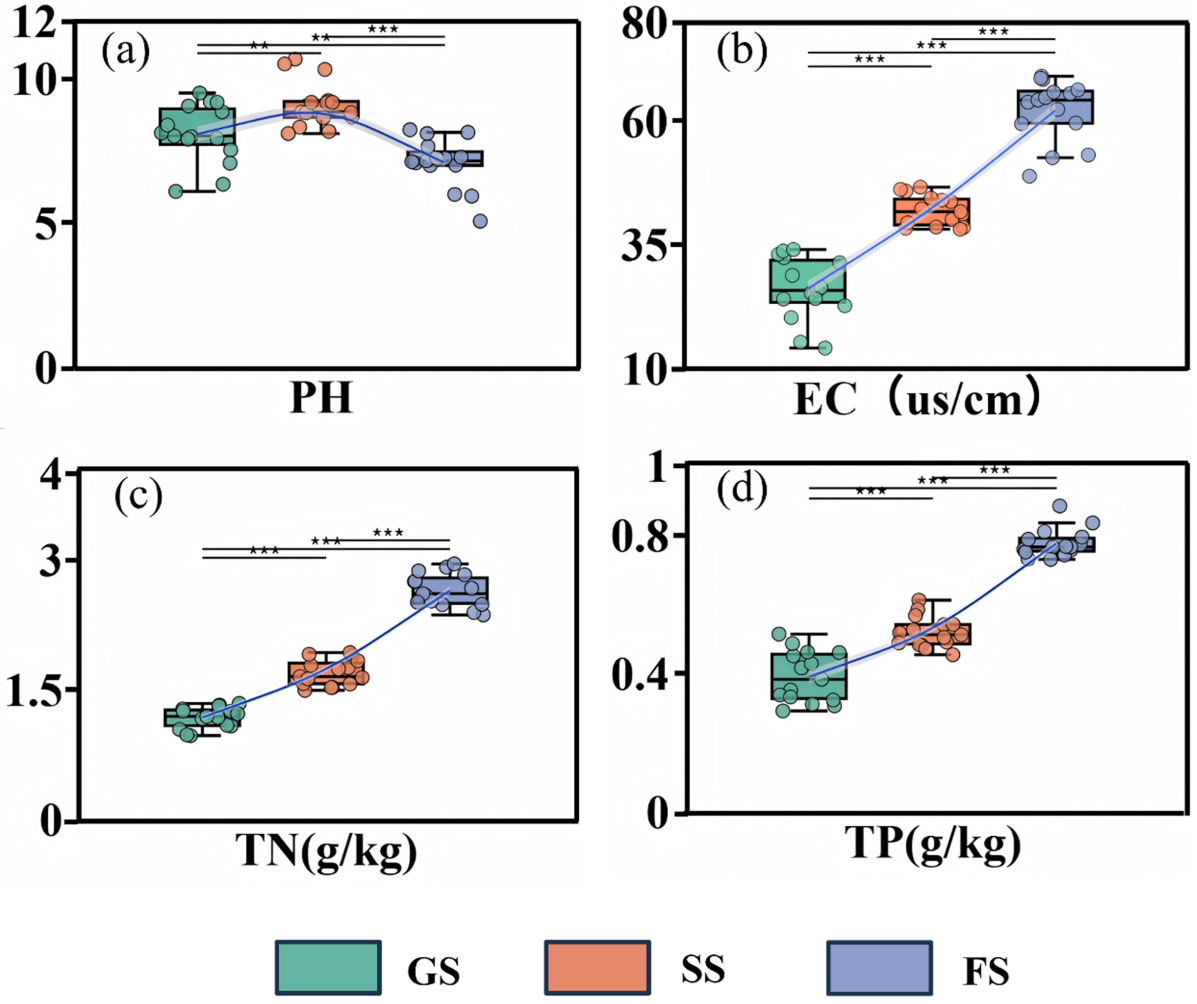
Soil physicochemical properties across vegetation succession stages in a karst ecosystem. (A) pH, (B) electrical conductivity (EC), (C) total nitrogen (TN), and (D) total phosphorus (TP). GS, grassland stage; SS, shrubland stage; FS, forest stage. Lines show trends across succession stages. Horizontal bars with asterisks indicate significant differences between stages. Significance: **P < 0.01, ***P < 0.001.

**Figure 3:**
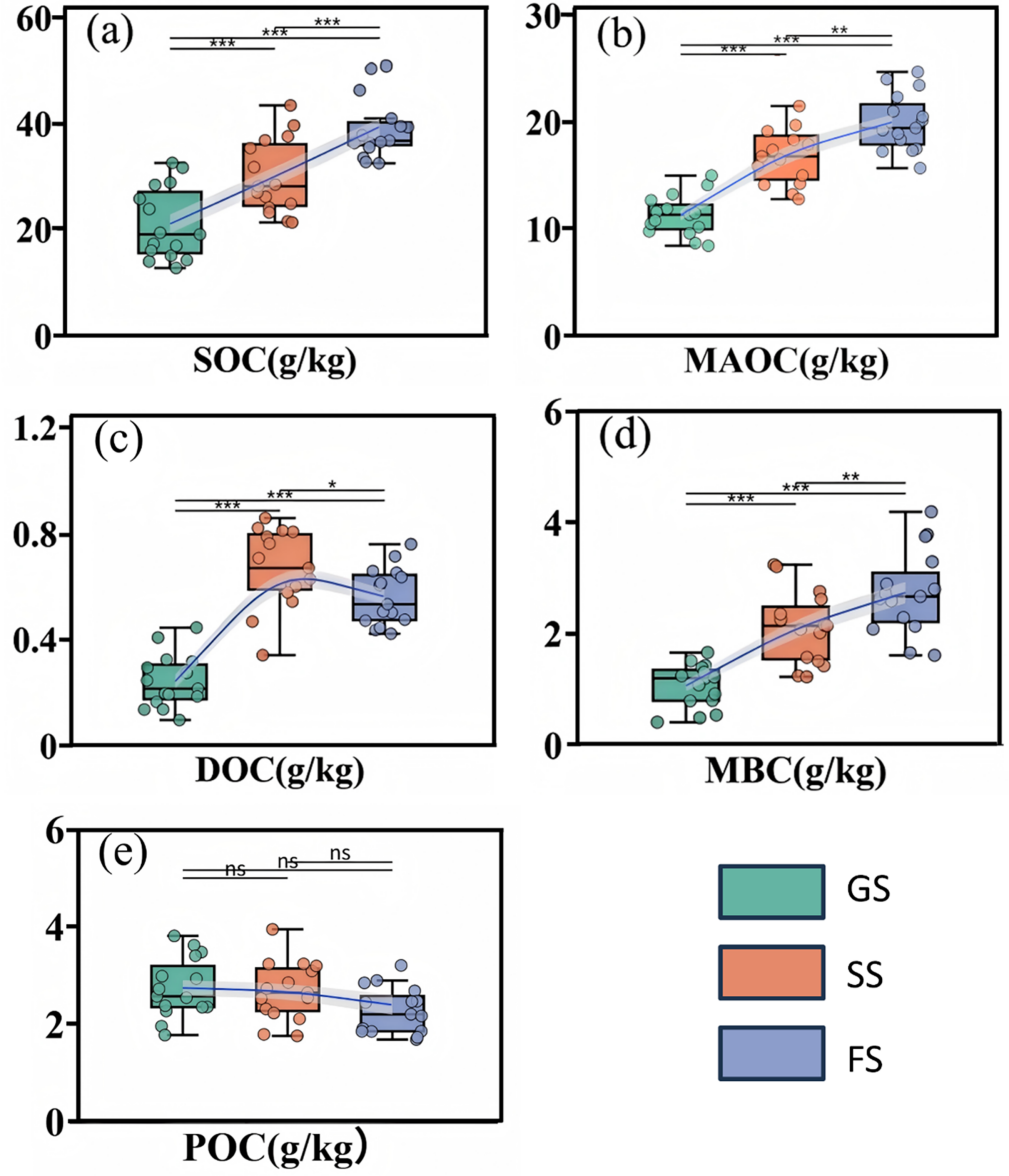
Soil organic carbon fractions across vegetation succession stages in a karst ecosystem. (A) soil organic carbon (SOC), (B) microbial biomass carbon (MBC), (C) dissolved organic carbon (DOC), (D) mineral-associated organic carbon (MAOC), and (E) particulate organic carbon (POC). GS, grassland stage; SS, shrubland stage; FS, forest stage. Lines show trends across succession stages. Horizontal bars with asterisks indicate significant differences between stages. Significance: ns *P* > 0.05, *P < 0.05, **P < 0.01, ***P < 0.001.

Vegetation regeneration succession significantly influenced soil physicochemical properties and soil carbon fractions (Figs. 2 and 3). Soil pH shifted from weakly alkaline in the grassland stage toward neutral in the forest stage, while electrical conductivity increased significantly (*P* < 0.05, Figs. 2A, 2B). Compared to the grassland stage, soil TN increased by 43.74% and 112.94% in the shrub and forest stages, respectively, while TP increased by 32.70% and 98.71% (Figs. 2C, 2D). Forest soil SOC content reached 39.45 ± 6.52 g/kg, representing increases of 76.74% and 52.32% over the grassland and shrub succession stages (Fig. 3A). With positive vegetation succession, soil MBC, DOC, and MAOC significantly increased (*P* < 0.05; Figs. 3B, 3C, 3D), with soil MBC showing the largest average increase of 63.84%. Soil POC exhibited no significant change (Fig. 3E).

#### Changes in the relative proportions of soil carbon fractions

During vegetation regeneration succession, the distribution proportions of soil MAOC, POC, DOC, and MBC within SOC varied. MAOC constituted the primary component of SOC, accounting for 51.76% to 58.82%; DOC exhibited the lowest proportion, ranging from 1.13% to 2.24%. As vegetation regeneration progressed, POC/SOC showed a significant decreasing trend (*P* < 0.05, Table 2); MBC/SOC was significantly higher in both the shrub and forest stages compared to the grassland stage (*P* < 0.05, Table 2); DOC/SOC reached its maximum value during the shrub stage (2.34 ± 0.74); while MAOC/SOC showed no significant change throughout the vegetation succession process (*P* > 0.05, Table 2).

**Table 2:** Changes in relative proportion of soil carbon components at different succession stages.

Succession stage	MAOC/SOC (%)	POC/SOC (%)	MBC/SOC (%)	DOC/SOC (%)
GS	57.60 ± 16.74a	14.21 ± 5.27a	9.48 ± 3.28b	1.13 ± 0.21a
SS	58.82 ± 10.19a	9.21 ± 2.07b	12.30 ± 2.93a	2.34 ± 0.74b
FS	51.76 ± 9.75a	6.30 ± 2.70c	14.03 ± 4.70a	1.48 ± 0.43a

**Note:**

Data in the table are the mean ± standard error, and different lowercase letters in the columns indicate variability in soil depth in the same community at the *P* < 0.05 level. MAOC/SOC is the ratio of mineral-associated organic carbon to soil organic carbon. POC/SOC is the ratio of particulate organic carbon to soil organic carbon. MBC/SOC is the ratio of microbial biomass carbon to soil organic carbon. DOC/SOC is the ratio of dissolved organic carbon to soil organic carbon.

### Characteristics of changes in soil extracellular enzyme activity during different stages of vegetation regeneration succession

**Figure 4:**
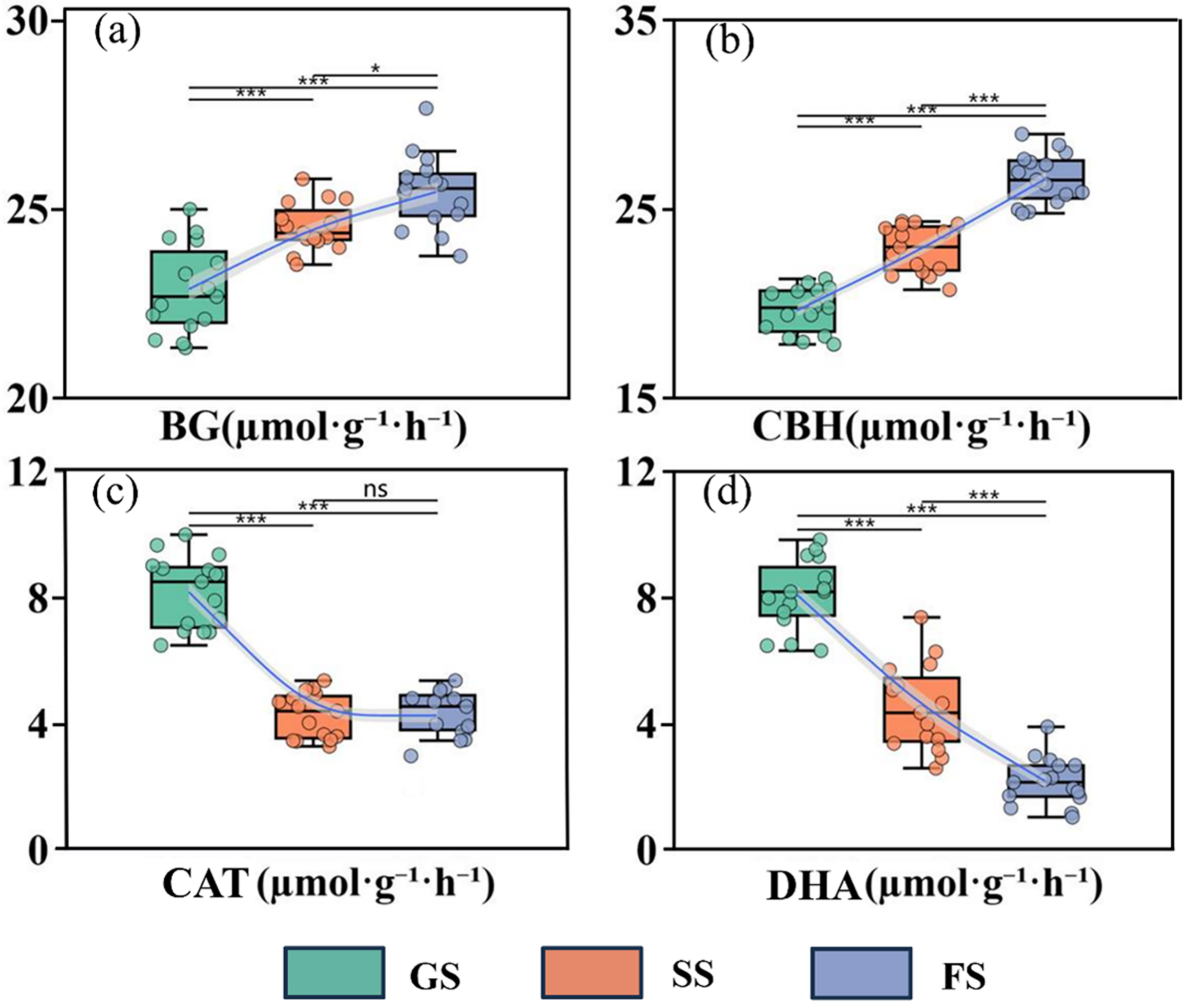
Soil extracellular enzyme activities across vegetation succession stages in a karst ecosystem. (A) β-1,4-glucosidase (BG), (B) cellobiohydrolase (CBH), (C) dehydrogenase (DHA), and (D) catalase (CAT). GS, grassland stage; SS, shrubland stage; FS, forest stage. Lines show trends across succession stages. Horizontal bars with asterisks indicate significant differences between stages. Significance: ns *P* > 0.05, *P < 0.05, ***P < 0.001.

As shown in Fig. 4, BG, CBH, CAT, and DHA exhibited distinct trends during vegetation regeneration succession. BG and CBH activities significantly increased with succession (*P* < 0.05), with CBH showing an average increase of 35.5%. Compared to the grassland stage, DHA activity decreased by 52.34% and 73.37% in the shrub and forest stages, respectively (*P* < 0.05). CAT activity peaked at 8.17 ± 1.15 μmol·g^−1^·h^−1^ during the grassland stage, with no significant differences observed between the shrub and forest stages (*P* > 0.05).

### Control analysis of SOC

**Figure 5:**
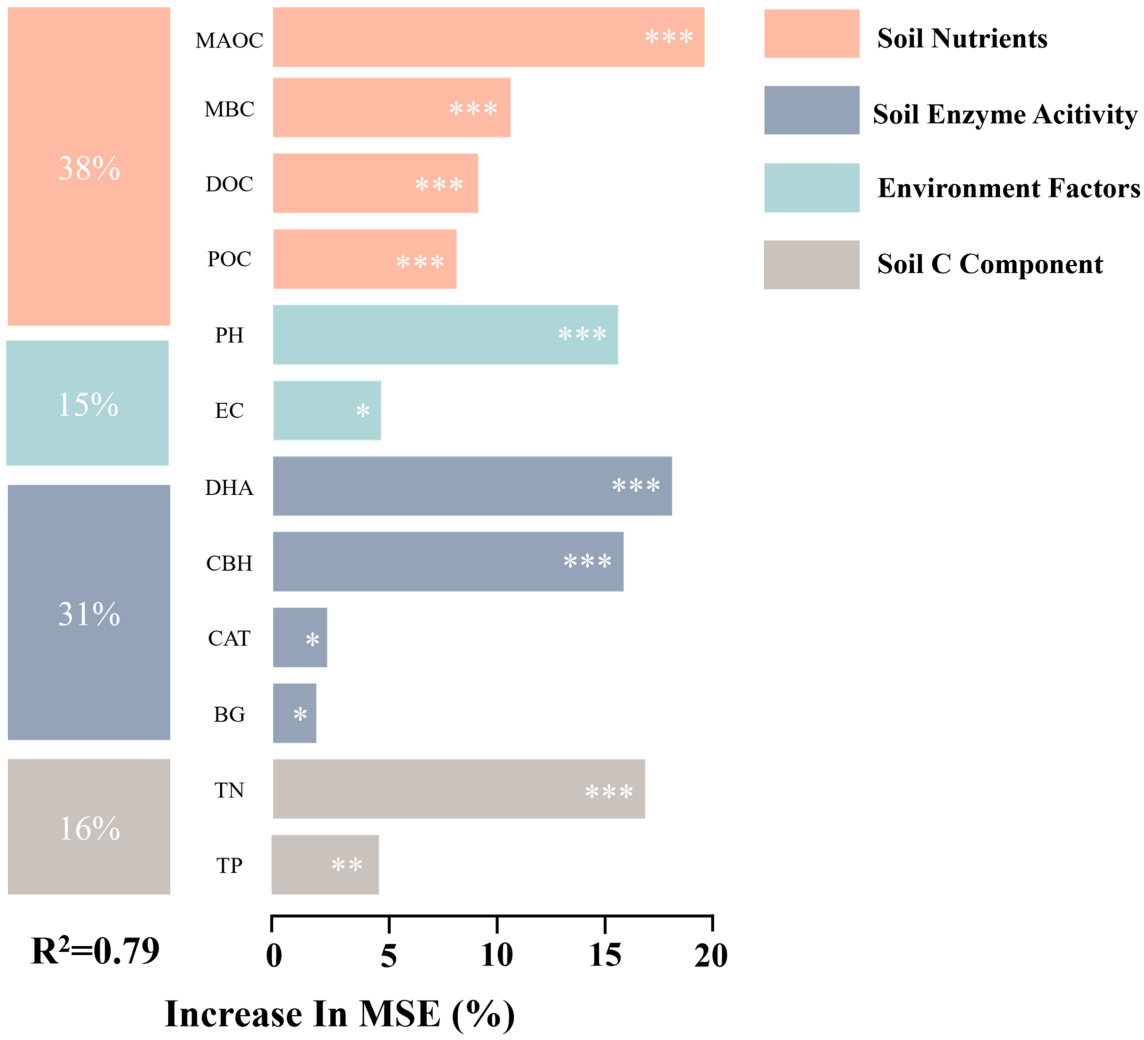
Random forest analysis of factors driving soil organic carbon variability. Based on a random forest regression model, representing the relative importance contribution of each factor to soil SOC; The bar length represents the importance of the variable (*i.e*., the percentage increase in mean square error calculated by the random forest model); Colors represent diûerent factor categories. Significance: *P < 0.05, **P < 0.01, ***P < 0.001.

**Figure 6:**
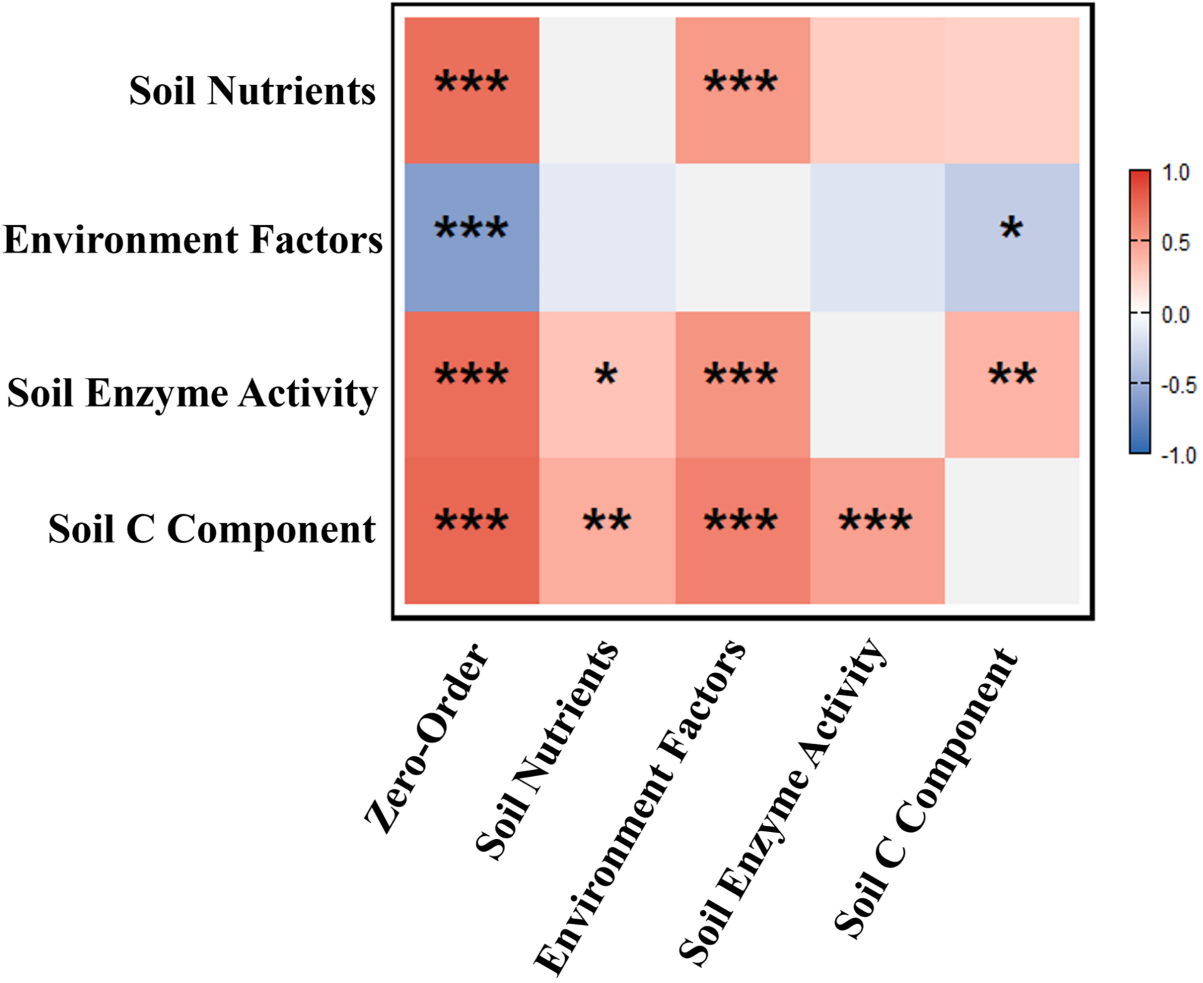
Correlations between soil organic carbon and key soil properties. Spearman correlation analysis between soil SOC and various factor groups; The depth of color represents the strength of correlation; Red represents positive correlation, blue represents negative correlation. Soil C components represent soil MAOC, MBC, DOC, and POC, soil nutrients represent soil TN and TP, environmental factors represent pH and EC, and soil enzyme activity represents BG, CBH, DHA, and CAT. Significance: ns *P* > 0.05, *P < 0.05, **P < 0.01, ***P < 0.001.

**Figure 7:**
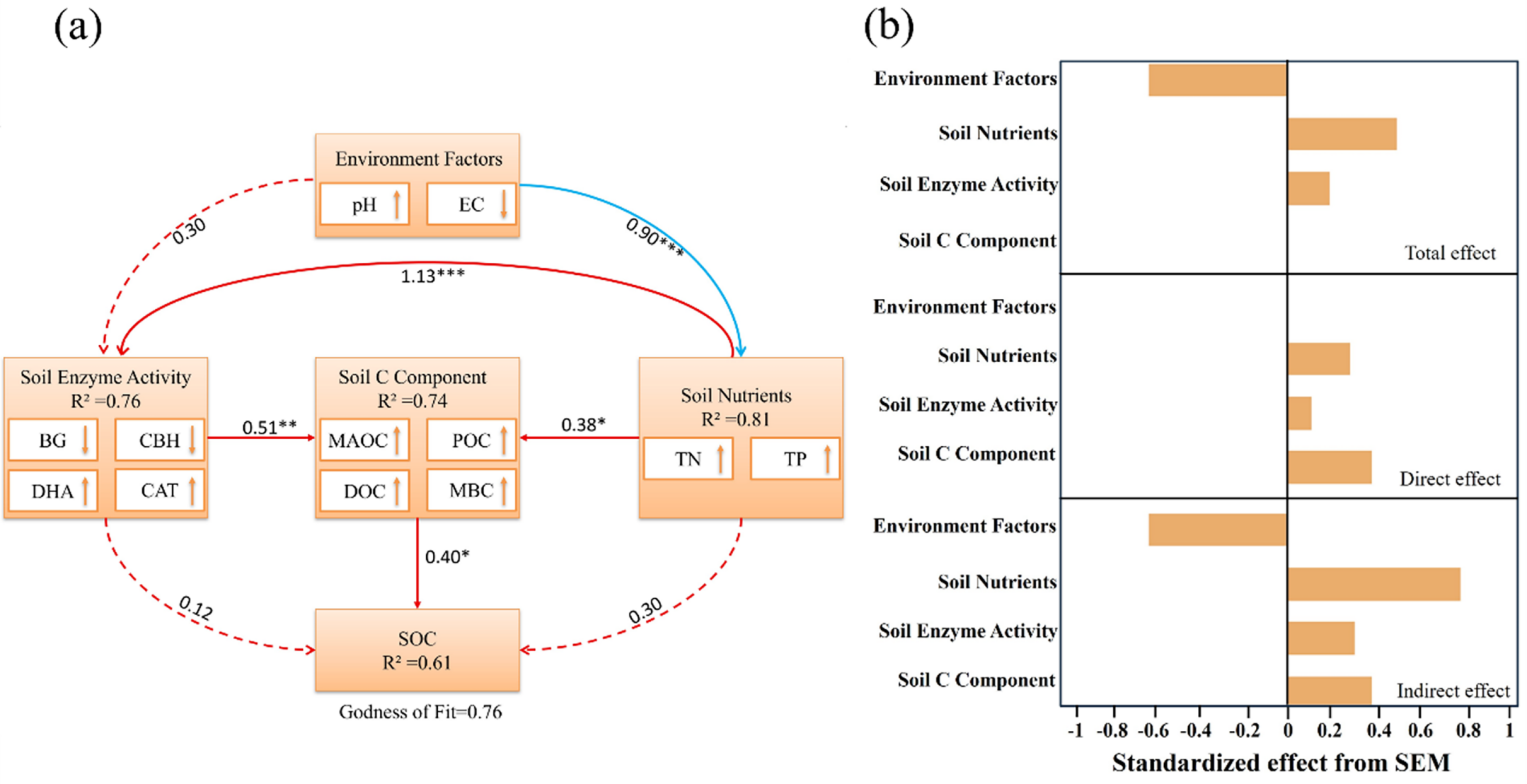
Path analysis of soil SOC based on partial least squares method. (A) Path analysis of SOC in vegetation regeneration succession. The red line represents positive effects, while the blue line represents negative effects. The solid line represents significant effects, the dashed line represents insignificant effects, and the thickness of the line reflects the absolute value of the path coefficient; The coefficient of determination (R^2^) of each factor group reflects the explanatory power of the model. (B) Representing the standardized effects of various factors on soil SOC, including total effects, direct effects, and indirect effects. Significance: *P < 0.05, **P < 0.01, ***P < 0.001.

As shown in Fig. 5, soil C component, soil nutrients, soil enzyme activity, and environment factors explained 38%, 31%, 16%, and 15% of the variance in SOC, respectively. Partial correlation analysis revealed that soil C component and soil enzyme activity significantly influenced SOC (Fig. 6). The effects of environment factors, soil enzyme activity, and soil C component on SOC were modulated by soil nutrients. After controlling for soil nutrients, their correlation coefficients with SOC decreased by 87%, 54%, and 44%, respectively. Path analysis results indicated that soil C component exerted the strongest direct effect on SOC, while soil nutrients had the greatest overall effect on SOC, primarily manifested as indirect effects (Fig. 7).

## Discussion

### Effects of vegetation regeneration succession on soil organic carbon and its components

During vegetation succession in karst regions, SOC content increased significantly (*P* < 0.05), with the trend following the order: GS < SS < FS, consistent with findings by Cheng et al. (2023), Liang et al. (2024). This increase was primarily driven by the progressive accumulation of litterfall and root exudates during succession (Breg Valjavec, Zorn & Čarni, 2018). In the early GS stage, herbaceous plants with shallow roots and low biomass limited both litterfall and root exudates, restricting SOC accumulation capacity (Zeng et al., 2024b). As succession advanced to the FS stage, nutrient release from decomposed litter significantly raised soil TN and TP content (*P* < 0.05), enhancing plant productivity and root development, which further increased organic carbon inputs (Hu et al., 2021). The developing root system also accelerated carbonate weathering, promoting SOC accumulation (Wen et al., 2021). H^+^ ions released during SOC accumulation contributed to a shift in soil pH from alkaline to neutral conditions. Similar trends have been observed in karst vegetation regeneration studies by Li et al. (2018), Pang et al. (2019). The neutralized soil environment favored soil enzyme activity, facilitating sustained SOC accumulation. Soil organic carbon fraction analysis revealed that soil MBC was most sensitive to vegetation succession, showing the greatest increase and peaking in the FS stage. The MBC/SOC ratio was significantly higher in the SS and FS stages than in the GS stage, indicating enhanced microbial carbon turnover efficiency (Yang et al., 2025). This increased microbial capacity to bind rhizosphere litter to mineral particles, significantly promoting MAOC accumulation and enhancing SOC stability. Soil POC content remained stable across succession stages, while MAOC increased significantly from grassland to shrubland to forest (*P* < 0.05). Although litter input increased in later stages, rapid microbial decomposition of POC offset new inputs, maintaining a dynamic equilibrium in POC levels (Kalinina et al., 2010). Microbial activity converted organic carbon into more stable MAOC, leading to a continuous increase in total SOC and a significant reduction in the POC/SOC ratio (Sokol & Bradford, 2019). While the MAOC/SOC ratio remained relatively stable, the contribution of POC to the total soil carbon pool diminished. Random forest analysis showed that MAOC was the primary contributor to SOC variability, indicating its key role in total SOC accumulation. In addition, soil DOC and the DOC/SOC ratio were higher in the SS stage. The relatively low vegetation cover at this stage made soil DOC more vulnerable to loss *via* surface runoff, partially explaining the limited SOC accumulation observed in the shrubland stage (Hu et al., 2025).

### Effects of vegetation regeneration succession on soil extracellular enzyme activity

Soil enzymes mediate the transformation of soil organic carbon through *in vitro* modification pathways, with distinct enzyme types exhibiting significant differences in their response characteristics to vegetation regeneration succession (Huang et al., 2024). This study indicates that BG and CBH activities significantly increased with vegetation regeneration succession (*P* < 0.05) (Fig. 3), consistent with findings by Tian et al. (2024). Vegetation succession enhances plant diversity, leading to litter accumulation and increased vegetation productivity, which in turn elevates soil organic carbon and nutrient content (Lange et al., 2015). This provides abundant material, energy, and nutrient resources for microbial activity during the forest stage, stimulating the physiological metabolism of tree roots to release more BG and CBH into the soil (Nardi et al., 2002). Furthermore, the higher DOC and MBC content in this stage further confirms that both the matrix and microorganisms can stimulate BG and CBH production and enhance their activity (Wu et al., 2025). Additionally, the suitable pH and higher electrical conductivity values in the forest stage optimize enzyme-substrate affinity, improving the catalytic efficiency of BG and CBH and significantly enhancing their activity (Luo et al., 2019). Increased BG and CBH activity promotes the conversion of litter to soil organic carbon, accelerating its accumulation. Conversely, CAT and DHA activities exhibit a declining trend during vegetation succession, with CAT activity stabilizing in the middle to late succession stages. CAT primarily decomposes hydrogen peroxide into water and oxygen, protecting microorganisms from oxidative damage (Bartkowiak & Lemanowicz, 2017). This may occur because pH tends toward neutrality during the mid-to-late succession phase, accompanied by increased SOC content. This indicates improved soil environmental conditions, reducing oxidative stress on microorganisms and allowing CAT activity to remain relatively stable. Notably, the unique karst geological conditions and shallow soil characteristics of the karst region studied here may exert significant influence on soil enzyme activity. The high calcium carbonate content in karst soils readily forms relatively compact soil structures, impairing soil aeration (Sakin & Gorawala, 2024). Concurrently, the generally thin soil layers in this region collectively limit the effective diffusion of atmospheric oxygen into the soil. Under relatively anoxic conditions, the metabolic activities of aerobic microorganisms are suppressed, consequently affecting the synthesis and active expression of aerobic enzymes such as CAT and DHA. DHA catalyzes the oxidative decomposition of organic matter and serves as a key indicator for assessing soil organic carbon mineralization activity (Kucerik et al., 2024). Its decreased activity indicates that as succession progresses, soil organic carbon mineralization intensity weakens, favoring SOC accumulation. This result aligns with the trend of gradually increasing soil organic carbon content during succession. Random forest analysis further confirms that DHA is a crucial enzyme influencing SOC accumulation.

### Regulatory mechanisms of soil organic carbon by soil nutrients, environmental factors, and enzyme activity

Research findings indicate that the compositional characteristics of soil carbon fractions directly determine the content and stability of SOC. Among these, carbon pools with varying stability directly constitute the material foundation of SOC. Active carbon fractions serve as the primary substrate for microbial metabolism, and changes in their content directly influence SOC turnover rates, while inert carbon fractions determine SOC’s long-term storage potential (Wang et al., 2023; Wu et al., 2024). Among these, soil MAOC constitutes the predominant fraction within the soil carbon pool and contributes most significantly to SOC variability. Due to its high stability, MAOC resists microbial decomposition and environmental disturbances (Georgiou et al., 2022). In calcium carbonate-rich karst soils, active sites on carbonate mineral surfaces promote organic carbon adsorption, facilitating MAOC formation and thereby enhancing soil carbon stability. Consequently, variations in MAOC content exert a significant direct influence on total SOC. SOC formation relies not only on the material basis of carbon components but also undergoes crucial regulation by soil nutrients. Further analysis using random forest, partial correlation, and path models revealed that SOC is primarily regulated by soil nutrients. On one hand, TN influences organic matter transformation processes by modulating enzyme activity (Gou et al., 2019). On the other hand, nitrogen inputs stimulate plant productivity, increasing organic carbon inputs (Reid et al., 2024). In contrast, TP exerts relatively weaker regulation on SOC. This disparity primarily stems from the differing chemical properties of nitrogen and phosphorus in karst soils: nitrogen predominantly exists in soluble forms, exhibiting high mobility and bioavailability; whereas phosphorus readily forms insoluble compounds with soil calcium carbonate, limiting its bioavailability (Grimm et al., 2024). Nevertheless, as a key element in energy transfer, TP indirectly influences soil organic carbon mineralization by regulating microbial energy metabolism (Ma et al., 2025). The significant increase in TN and TP content during vegetation regeneration succession further validates the promotional effect of improved nutrient status on soil carbon sequestration (Wang et al., 2022). Furthermore, environmental factors exert negative effects on SOC. Partial correlation analysis indicates these negative effects primarily act indirectly on SOC by influencing nutrient availability. The shift toward neutral pH alters the chemical transformation of nitrogen and phosphorus elements, while increased electrical conductivity reflects a high ionic strength environment. This, in turn, reduces the bioavailability of TN and TP through ion competition effects (Xie et al., 2025; Peng et al., 2025). The resulting decline in nutrient availability further restricts microbial activity, thereby explaining the indirect mechanism through which environmental factors exert negative effects on SOC.

## Conclusions

This study elucidates the mechanisms through which vegetation regeneration succession enhances soil organic carbon (SOC) sequestration in southwest China’s karst regions, confirming that long-term regeneration significantly improves carbon sink function in this fragile ecosystem. Our findings reveal that mineral-bound organic carbon (MAOC) dominates karst soil carbon pools and determines long-term stability, while microbial biomass carbon (MBC) drives carbon pool transformation. Soil extracellular enzymes exhibit adaptive responses during regeneration, with enhanced β-1,4-glucosidase and cellulase activities promoting carbon input and reduced dehydrogenase and catalase activities facilitating accumulation. Mechanistic analysis demonstrates that soil carbon fractions are the primary drivers of SOC variability, with nutrients (particularly total nitrogen) and environmental factors (pH and electrical conductivity) exerting indirect regulatory effects through their influence on enzyme activities and microbial processes. These findings provide critical scientific evidence for optimizing ecological regeneration strategies and carbon sink management in karst regions, offering important implications for regional ecosystem recovery and achieving carbon neutrality goals. Nevertheless, soil–bedrock heterogeneity in karst landscapes and the relatively limited sample size may introduce uncertainties in cross-stage comparisons, warranting validation through long-term field monitoring.

## Supplemental Information

10.7717/peerj.21125/supp-1 Supplemental Information 1 Data.

## Additional Information and Declarations

### Data Availability

The following information was supplied regarding data availability:

The raw data is available in the Supplemental File.

## References

[ref-1] Araújo ASF, Cesarz S, Leite LFC, Borges CD, Tsai SM, Eisenhauer N (2013). Soil microbial properties and temporal stability in degraded and restored lands of Northeast Brazil. Soil Biology and Biochemistry.

[ref-2] Bao S (2020). Soil Agro-chemistrical Analysis.

[ref-3] Bartkowiak A, Lemanowicz J (2017). Effect of forest fire on changes in the content of total and available forms of selected heavy metals and catalase activity in soil. Soil Science Annual.

[ref-4] Bastin J-F, Finegold Y, Garcia C, Mollicone D, Rezende M, Routh D, Zohner CM, Crowther TW (2019). The global tree restoration potential. Science.

[ref-5] Bossio DA, Cook-Patton SC, Ellis PW, Fargione J, Sanderman J, Smith P, Wood S, Zomer RJ, von Unger M, Emmer IM, Griscom BW (2020). The role of soil carbon in natural climate solutions. Nature Sustainability.

[ref-6] Breg Valjavec M, Zorn M, Čarni A (2018). Bioindication of human-induced soil degradation in enclosed karst depressions (dolines) using Ellenberg indicator values (Classical Karst, Slovenia). Science of the Total Environment.

[ref-7] Breg Valjavec M, Čarni A, Žlindra D, Zorn M, Marinšek A (2022). Soil organic carbon stock capacity in Karst dolines under different land uses. Catena.

[ref-8] Burns RG, DeForest JL, Marxsen J, Sinsabaugh RL, Stromberger ME, Wallenstein MD, Weintraub MN, Zoppini A (2013). Soil enzymes in a changing environment: current knowledge and future directions. Soil Biology and Biochemistry.

[ref-9] Carrascal LM, Galván I, Gordo O (2009). Partial least squares regression as an alternative to current regression methods used in ecology. Oikos.

[ref-10] Cheng H, Zhou X, Dong R, Wang X, Liu G, Li Q (2023). Natural vegetation regeneration facilitated soil organic carbon sequestration and microbial community stability in the degraded karst ecosystem. Catena.

[ref-11] Dixon RK, Brown S, Houghton RA, Solomon AM, Trexler MC, Wisniewski J (1994). Carbon pools and flux of global forest ecosystems. Science.

[ref-12] Dunn L, Lang C, Marilleau N, Terrat S, Biju-Duval L, Lelièvre M, Perrin S, Chemidlin Pré¬vost-Bouré N (2021). Soil microbial communities in the face of changing farming practices: a case study in an agricultural landscape in France. PLOS ONE.

[ref-13] Fanin N, Mooshammer M, Sauvadet M, Meng C, Alvarez G, Bernard L, Bertrand I, Blagodatskaya E, Bon L, Fontaine S, Niu S, Lashermes G, Maxwell TL, Weintraub MN, Wingate L, Moorhead D, Nottingham AT (2022). Soil enzymes in response to climate warming: mechanisms and feedbacks. Functional Ecology.

[ref-14] Georgiou K, Jackson RB, Vindušková O, Abramoff RZ, Ahlström A, Feng W, Harden JW, Pellegrini AFA, Polley HW, Soong JL, Riley WJ, Torn MS (2022). Global stocks and capacity of mineral-associated soil organic carbon. Nature Communications.

[ref-15] Gou X, Hu J, Chen Y, Wei X, Du Z, Zhou Q, Zhou Q (2019). The effect of artificial vegetation recovery on the soil nutrients and enzyme activities in subhumid desert land on the southeast Qinghai-Tibetan Plateau, China. Ecological Engineering.

[ref-16] Grimm H, Drabesch S, Nicol A, Straub D, Joshi P, Zarfl C, Planer-Friedrich B, Muehe EM, Kappler A (2024). Arsenic immobilization and greenhouse gas emission depend on quantity and frequency of nitrogen fertilization in paddy soil. Heliyon.

[ref-17] Guo Z, Zhang X, Green SM, Dungait JAJ, Wen X, Quine TA (2019). Soil enzyme activity and stoichiometry along a gradient of vegetation restoration at the Karst Critical Zone Observatory in Southwest China. Land Degradation & Development.

[ref-18] He G, Liu X, Li Y, Xu H, Ji T, Yang Z, Qi H, Ma C, Wang Y, Zhang D, Lin D, Shi Y, Jiang J (2024). Recovery in soil carbon stocks but reduced carbon stabilization after near-natural restoration in degraded alpine meadows. Scientific Reports.

[ref-19] Hu X, Li Y, Guo W, Li J, Zhu X, Luo L, Tian P, Sun H (2025). Inhibiting effects of vegetation coverage on runoff, sediment, and organic carbon loss on steep loess gully-slopes. Land Degradation & Development.

[ref-20] Hu P, Zhang W, Chen H, Li D, Zhao Y, Zhao J, Xiao J, Wu F, He X, Luo Y, Wang K (2021). Soil carbon accumulation with increasing temperature under both managed and natural vegetation restoration in calcareous soils. Science of the Total Environment.

[ref-21] Huang Q, Wang B, Shen J, Xu F, Li N, Jia P, Jia Y, An S, Amoah ID, Huang Y (2024). Shifts in C-degradation genes and microbial metabolic activity with vegetation types affected the surface soil organic carbon pool. Soil Biology and Biochemistry.

[ref-22] Huang X, Zhang Z, Zhou Y, Wang X, Zhang J, Zhou X (2021). Characteristics of soil organic carbon under different karst landforms. Carbonates and Evaporites.

[ref-23] Jiang PK, Xu QF, Xu ZH, Cao ZH (2006). Seasonal changes in soil labile organic carbon pools within a Phyllostachys praecox stand under high rate fertilization and winter mulch in subtropical China. Forest Ecology and Management.

[ref-24] Kalinina O, Goryachkin SV, Karavaeva NA, Lyuri DI, Giani L (2010). Dynamics of carbon pools in post-agrogenic sandy soils of southern taiga of Russia. Carbon Balance and Management.

[ref-25] Kucerik J, Brtnicky M, Mustafa A, Hammerschmiedt T, Kintl A, Sobotkova J, Alamri S, Baltazar T, Latal O, Naveed M, Malicek O (2024). Utilization of diversified cover crops as green manure-enhanced soil organic carbon, nutrient transformation, microbial activity, and maize growth. Agronomy.

[ref-26] Lange M, Eisenhauer N, Sierra CA, Bessler H, Engels C, Griffiths RI, Mellado-Vázquez PG, Malik AA, Roy J, Scheu S, Steinbeiss S, Thomson BC, Trumbore SE, Gleixner G (2015). Plant diversity increases soil microbial activity and soil carbon storage. Nature Communications.

[ref-27] Li P (2018). Guizhou launches remote sensing monitoring in the FAST electromagnetic wave quiet zone. China Science Daily.

[ref-29] Li D, Wen L, Jiang S, Song T, Wang K (2018). Responses of soil nutrients and microbial communities to three restoration strategies in a karst area, southwest China. Journal of Environmental Management.

[ref-30] Li S, Yan C, Zhu M, Yan S, Wang J, Qian F (2025). Response patterns of soil organic carbon fractions and storage to vegetation types in the Yellow River Wetland. Land.

[ref-31] Liang Y, Fu R, Sailike A, Hao H, Yu Z, Wang R, Peng N, Li S, Zhang W, Liu Y (2024). Soil labile organic carbon and nitrate nitrogen are the main factors driving carbon-fixing pathways during vegetation restoration in the Loess Plateau, China. Agriculture, Ecosystems and Environment.

[ref-32] Liu X, Huang X, Qin W, Li X, Ma Z, Shi H, Li L, Li C (2022). Effects of establishing cultivated grassland on soil organic carbon fractions in a degraded alpine meadow on the Tibetan Plateau. PeerJ.

[ref-33] Liu S, Zhang W, Wang K, Pan F, Yang S, Shu S (2015). Factors controlling accumulation of soil organic carbon along vegetation succession in a typical karst region in Southwest China. Science of the Total Environment.

[ref-34] Luo X, Hou E, Zhang L, Zang X, Yi Y, Zhang G, Wen D (2019). Effects of forest conversion on carbon-degrading enzyme activities in subtropical China. Science of the Total Environment.

[ref-35] Ma X, Li X, Meng Y, Liu J, Wang J, Yu X, Wang W, Xu X (2025). Influence of soil depth and land use type on the diversity of and metabolic restriction in the soil microbial community of a forest-grass ecotone. Microorganisms.

[ref-36] Mir YH, Ganie MA, Shah TI, Aezum AM, Bangroo SA, Mir SA, Dar SR, Mahdi SS, Baba ZA, Shah AM, Majeed U, Minkina T, Rajput VD, Dar AA (2023). Soil organic carbon pools and carbon management index under different land use systems in North Western Himalayas. PeerJ.

[ref-37] Nan R, Jiang P (2017). 500-meter aperture spherical radio telescope (FAST). Journal of Mechanical Engineering.

[ref-38] Nannipieri P, Giagnoni L, Renella G, Puglisi E, Ceccanti B, Masciandaro G, Fornasier F, Moscatelli MC, Marinari S (2012). Soil enzymology: classical and molecular approaches. Biology and Fertility of Soils.

[ref-39] Nardi S, Pizzeghello D, Muscolo A, Vianello A (2002). Physiological effects of humic substances on higher plants. Soil Biology and Biochemistry.

[ref-40] Nelson DW, Sommers LE (1996). Total carbon, organic carbon, and organic matter. Methods of soil analysis.

[ref-41] Ojha RB, Kristiansen P, Atreya K, Wilson B (2023). Changes in soil organic carbon fractions in abandoned croplands of Nepal. Geoderma Regional.

[ref-42] Pan Y, Birdsey RA, Fang J, Houghton R, Kauppi PE, Kurz WA, Phillips OL, Shvidenko A, Lewis SL, Canadell JG, Ciais P, Jackson RB, Pacala SW, McGuire AD, Piao S, Rautiainen A, Sitch S, Hayes D (2011). A large and persistent carbon sink in the world’s forests. Science.

[ref-43] Pang D, Cui M, Liu Y, Wang G, Cao J, Wang X, Dan X, Zhou J, Li Y (2019). Responses of soil labile organic carbon fractions and stocks to different vegetation restoration strategies in degraded karst ecosystems of southwest China. Ecological Engineering.

[ref-44] Peng N, Wang Y, Wu H, Hao H, Sailike A, Yu Z, Li S, Shi R, Hao W, Zhang W (2025). Phosphorus cycling dominates microbial regulation of synergistic carbon, nitrogen, and phosphorus gene dynamics during Robinia pseudoacacia restoration on the Loess Plateau. Agronomy.

[ref-45] R Core Team (2024). R: a language and environment for statistical computing.

[ref-46] Reid NM, Wigley K, Nusrath A, Smaill SJ, Garrett LG (2024). Use of nitrogen-fixing plants to improve planted forest soil fertility and productivity in New Zealand: a review. New Zealand Journal of Forestry Science.

[ref-47] Sakin E, Gorawala P, Gorawala P, Mandhatri S (2024). The advantages and disadvantages of calcareous soils. Agricultural Research Updates.

[ref-48] Shi D (2014). Performance and sustainable development of the Grain-for-Green program in Pingtang County, Guizhou Province. Beijing Agriculture.

[ref-49] Shi J, Song M, Yang L, Zhao F, Wu J, Li J, Yu Z, Li A, Shangguan Z, Deng L (2023). Recalcitrant organic carbon plays a key role in soil carbon sequestration along a long-term vegetation succession on the Loess Plateau. Catena.

[ref-50] Shi K, Sun Y, Zeng D-H, Sheng Z, Zhang Y, Lin G (2025). Oxidative enzymes underlie tree species effects on soil organic carbon stocks: a common garden test with eight tree species. Soil Biology and Biochemistry.

[ref-51] Sokol N, Bradford MA (2019). Microbial formation of stable soil carbon is more efficient from belowground than aboveground input. Nature Geoscience.

[ref-52] Sollins P, Swanston C, Kramer M (2007). Stabilization and destabilization of soil organic matter—a new focus. Biogeochemistry.

[ref-53] Suleymanov A, Gabbasova I, Komissarov M, Suleymanov R, Garipov T, Tuktarova I, Belan L (2023). Random forest modeling of soil properties in saline semi-arid areas. Agriculture.

[ref-54] Tian Q, Yang F, Wang Z, Zhang Q (2024). Variation of soil organic carbon components and enzyme activities during the ecological restoration in a temperate forest. Ecological Engineering.

[ref-55] Vance ED, Brookes PC, Jenkinson DS (1987). An extraction method for measuring soil microbial biomass carbon. Soil Biology and Biochemistry.

[ref-56] Wang L, Luo N, Shi Q, Sheng M (2023). Responses of soil labile organic carbon fractions and enzyme activities to long-term vegetation restorations in the karst ecosystems, Southwest China. Ecological Engineering.

[ref-57] Wang H, Wu J, Li G, Yan L (2020). Changes in soil carbon fractions and enzyme activities under different vegetation types of the northern Loess Plateau. Ecology and Evolution.

[ref-59] Wang Z, Zhou M, Liu H, Huang C, Ma Y, Ge H, Ge X, Fu S (2022). Pecan agroforestry systems improve soil quality by stimulating enzyme activity. PeerJ.

[ref-60] Wani SA (2021). Assessment of changes in soil organic carbon fractions and enzyme activities under apple growing ecosystems in temperate North-Western Himalayas. Resources, Environment and Sustainability.

[ref-61] Wen H, Sullivan PL, Macpherson GL, Billings SA, Li L (2021). Deepening roots can enhance carbonate weathering by amplifying CO2-rich recharge. Biogeosciences.

[ref-62] Wu X, Peng Y, Wang T, Ahmad N, Bai X, Chang R (2025). Distinct responses of soil carbon-degrading enzyme activities to warming in two alpine meadow ecosystems on the Qinghai-Tibet Plateau. Soil Ecology Letters.

[ref-63] Wu B, Zhang M, Zhai Z, Dai H, Yang M, Zhang Y, Liang T (2024). Soil organic carbon, carbon fractions, and microbial community under various organic amendments. Scientific Reports.

[ref-64] Xie Z, Zhao R, Bo B, Li C, Wang Y, Chu Y, Ye C (2025). Effect of crop type shift on soil phosphorus morphology and microbial functional diversity in a typical Yellow River irrigation area. Microorganisms.

[ref-65] Xu H, Qu Q, Liu G, Xue S, Li P (2020). Response of soil specific enzyme activity to vegetation restoration in the Loess hilly region of China. Catena.

[ref-66] Yang Y, Gunina A, Cheng H, Liu L, Wang B, Dou Y, Wang Y, Liang C, An S, Chang SX (2025). Unlocking mechanisms for soil organic matter accumulation: carbon use efficiency and microbial necromass as the keys. Global Change Biology.

[ref-67] Yang F, Zhong Y, Han G, Li X, Luo L, Cai X, Long X, Li T, Huang L (2023). Effect of different vegetation restoration on soil organic carbon dynamics and fractions in the Rainy Zone of Western China. Journal of Environmental Management.

[ref-68] Zeng C, Li TY, He BH, Feng MD, Liang K (2024a). Vegetation succession increases soil organic carbon density and decreases soil erodibility: evidence from a karst trough valley experiencing farmland abandonment. Catena.

[ref-69] Zeng J, Li X, Jian J, Xing L, Li Y, Wang X, Zhang Q, Ren C, Yang G, Han X (2024b). Differences in the regulation of soil carbon pool quality and stability by leaf-litter and root-litter decomposition. Environmental Research.

[ref-70] Zhang L, Du H, Song T, Yang Z, Peng W, Gong J, Huang G, Li Y (2024a). Conversion of farmland to forest or grassland improves soil carbon, nitrogen, and ecosystem multi-functionality in a subtropical karst region of southwest China. Scientific Reports.

[ref-71] Zhang Y, Tang Z, You Y, Guo X, Wu C, Liu S, Sun OJ (2023). Differential effects of forest-floor litter and roots on soil organic carbon formation in a temperate oak forest. Soil Biology and Biochemistry.

[ref-72] Zhang Y, Wang X, Zheng Y, Duan L (2024c). Characteristics of soil organic carbon fractions and influencing factors in different understory mosses in karst urban parks. Scientific Reports.

[ref-73] Zhang J, Zhang T, Pu L, Yan L, Cai G, Chen P, Yang T, Zhang C (2022). Litter eco-hydrological function characteristics of three typical plant communities in the area of Karst peak-cluster depressions from Guizhou, China. PLOS ONE.

[ref-74] Zhang S, Zhou X, Chen Y, Du F, Zhu B (2024b). Soil organic carbon fractions in China: spatial distribution, drivers, and future changes. Science of the Total Environment.

